# Supplementary material for: Investigating the influence of physiologically relevant hydrostatic pressure on CHO cell batch culture
Source: Sci Rep. 2021 Jan 8;11:162. doi: 10.1038/s41598-020-80576-8 (PMC7794228; doi:10.1038/s41598-020-80576-8)
Supplement: Supplementary file 1 — Supplementary Information 1. [file 41598_2020_80576_MOESM1_ESM.zip › S1/IgG/Feb17 60 90 standard/standard/60ugml volume.pdf]

Data File C:\Users\P...s\ChemStation\1\Data\TK_CHO_IgG 2019-02-17 17-28-56\009-6-60ug_ml.D
Sample Name: 60ug/ml

===

Acq. Operator : SYSTEM   Seq. Line : 9
Sample Operator : SYSTEM
Acq. Instrument : HPLC-0039   Location : 6
Injection Date : 2/17/2019 6:04:38 PM   Inj : 1
Inj Volume : 100.000 µl
Different Inj Volume from Sample Entry! Actual Inj Volume : 60.000 µl
Sequence File : C:\Users\Public\Documents\ChemStation\1\Data\TK_CHO_IgG 2019-02-17 17-28-56\TK_CHO_IgG.S
Method : C:\Users\Public\Documents\ChemStation\1\Data\TK_CHO_IgG 2019-02-17 17-28-56\CHO_IgG_LC.M (Sequence Method)
Last changed : 2/8/2019 3:17:23 PM by SYSTEM

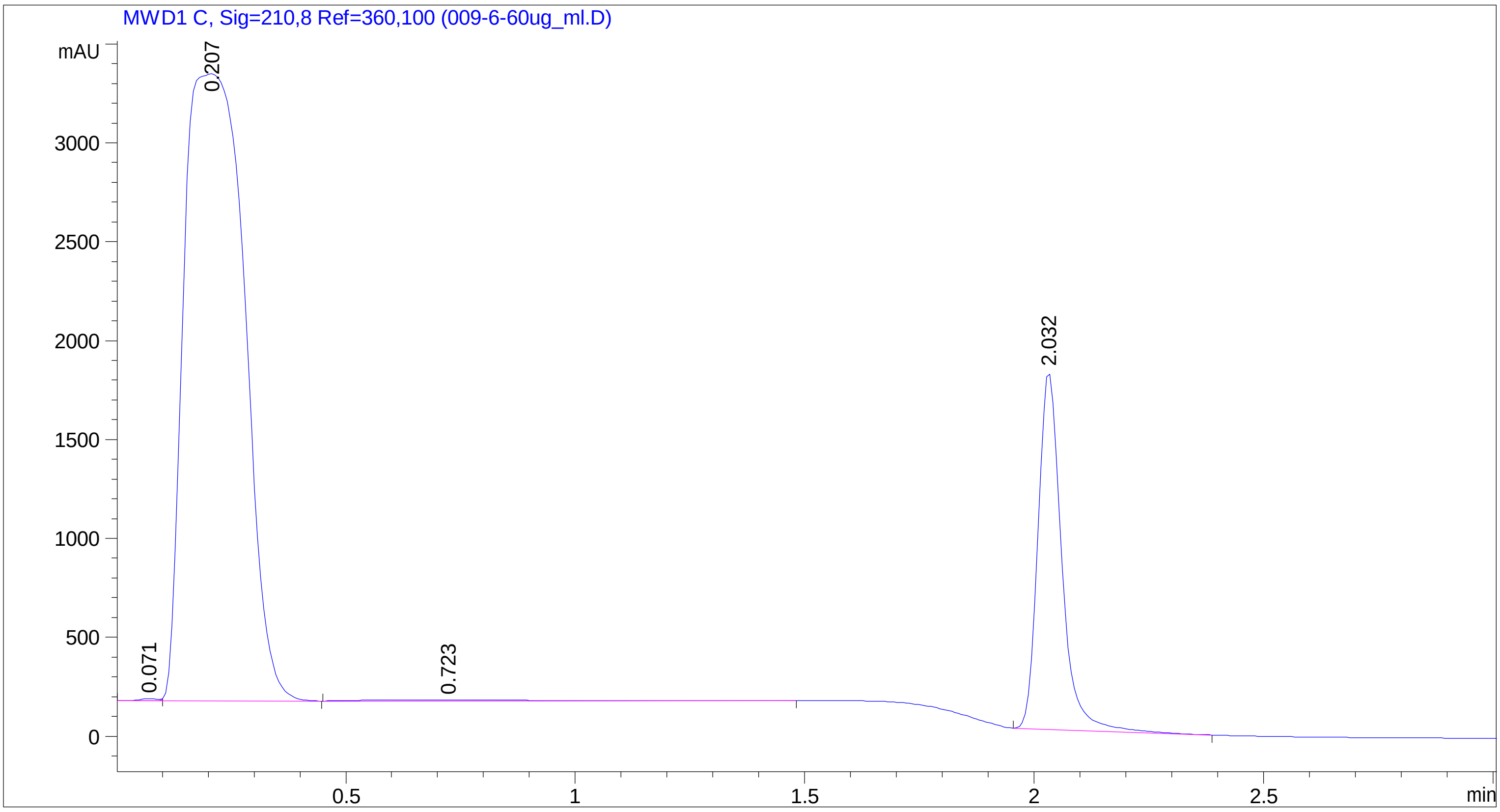

===

Area Percent Report

===

Sorted By : Signal
Multiplier : 1.0000
Dilution : 1.0000
Use Multiplier & Dilution Factor with ISTDs

Signal 1: MWD1 C, Sig=210,8 Ref=360,100

Peak #	RetTime [min]	Type	Width [min]	Area [mAU*s]	Height [mAU]	Area %
1	0.071	BV E	0.0396	29.49963	11.61289	0.0829
2	0.207	VB R	0.1344	2.89210e4	3170.45142	81.2582
3	0.723	BB	0.3545	136.71620	4.62954	0.3841
4	2.032	BBA	0.0539	6504.27393	1808.98059	18.2748

Totals : 3.55915e4 4995.67444

===

*** End of Report ***
